# TNFAIP8 promotes prostate cancer cell survival by inducing autophagy

**DOI:** 10.18632/oncotarget.25529

**Published:** 2018-06-01

**Authors:** Suryakant Niture, Malathi Ramalinga, Habib Kedir, Dorrelyn Patacsil, Samiksha S. Niture, James Li, Haresh Mani, Simeng Suy, Sean Collins, Deepak Kumar

**Affiliations:** ^1^ Julius L. Chambers Biomedical Biotechnology Research Institute, North Carolina Central University Durham, 27707 NC, USA; ^2^ Cancer Research Laboratory, University of the District of Columbia, Washington, 20008 DC, USA; ^3^ Catonsville High School, Catonsville, 21228 MD, USA; ^4^ Lombardi Comprehensive Cancer Center, Georgetown University, Washington, 20008 DC, USA; ^5^ Department of Pathology, Inova Fairfax Hospital, Falls Church, 22042 VA, USA

**Keywords:** TNFAIP8, autophagy, cell survival, prostate cancer, neuroendocrine differentiation

## Abstract

Tumor necrosis factor-α-inducible protein 8 (TNFAIP8) is a TNF-α inducible anti-apoptotic protein with multiple roles in tumor growth and survival. Mechanisms of cell survival by TNFAIP8 remain elusive. We investigated the role of TNFAIP8 in the regulation of the cell cycle, autophagy, cell survival and neuroendocrine differentiation in prostate cancer cells. We showed that TNFAIP8 dysregulates cell-cycle-related proteins, in PC3 cells. Oncogenic cell survival, drug resistance and dysregulation of cell cycle-related proteins are often associated with autophagy. We demonstrated that TNFAIP8 induces autophagy by increasing expression of autophagy effectors such as LC3β I/II, Beclin1, 4EBP1, p62, and SIRT1. We also demonstrated that TNFAIP8 interacts with autophagy-related protein 3 (ATG3). TNFα treatment increased the expression of TNFAIP8, which was associated with increased autophagy and decreased apoptosis. We also observed an increase in expression of neuroendocrine differentiation markers, synaptophysin and chromogranin A, and drug resistance to anticancer drugs, docetaxel and doxorubicin, in cells transfected with TNFAIP8. Collectively, our findings reveal that by the creation of cellular autophagy events, TNFAIP8 promotes cell survival and drug resistance in prostate cancer cells.

## INTRODUCTION

Tumor necrosis factor α-inducible protein 8 (TNFAIP8; also known as SCC-S2, GG2-1, and NDED) is a TNFα inducible protein. TNFAIP8 family proteins were initially identified by comparing two matched primary and metastatic head and neck squamous cell carcinoma cell lines [1] and by investigating a TNFα inducible gene in endothelial cells [2]. The TNFAIP8 family includes TNFAIP8, TNFAIP8-like protein 1 (TIPE1), TNFAIP8-like protein 2 (TIPE2), and TNFAIP8-like protein 3 (TIPE3) proteins [3–5]. Several isoforms of TNFAIP8 have been identified which are involved in various physiological processes and diseases, including cancer [5–7]. TNFAIP8 is induced by NF-kB, inhibits cellular apoptosis, acts as an oncogenic molecule, and promotes cell growth/proliferation in human cancers [6, 8–11].

TNFAIP8 contains a death effector domain, which negatively regulates apoptosis in several types of cancer [4]. Depletion of TNFAIP8 in tumor cells has been shown to increase expression of genes associated with proliferation suppression, apoptosis, and fatty-acid oxidation genes and to decrease expression of several oncogenes [12]. TNFAIP8 v2 can promote cancer by broadly repressing p53 function [13]. Silencing of TNFAIP8 v2 in cancer cells induces p53-independent inhibition of DNA synthesis and widespread p53 binding. TNFAIP8 v2 silencing also upregulates target genes, initiates p53-dependent cell-cycle arrest, and sensitizes cells to DNA damage [13]. These studies suggest that TNFAIP8 negatively regulates apoptosis and promotes cell growth.

Recently, Sun, *et al*. [14] showed that TNFAIP8-deficient mice had increased colitis severity compared with wild-type mice due to the lack of TNFAIP8 expression in non-hemopoietic cells. Furthermore, intestinal epithelial cells from TNFAIP8-deficient mice had higher cell-death rates and decreased proliferation compared with those from wild-type mice. TNFAIP8 inhibits Ras-related C3 botulinum toxin substrate 1 (RAC1), which regulates bacterial *Listeria monocytogenes* infections by controlling pathogen invasion and host-cell apoptosis [15]. In that study, TNFAIP8-knockout mice were resistant to lethal *L. monocytogenes* infection and had a decreased bacterial load in the liver and spleen [15]. In Drosophila, a loss-of-function mutation in the TNFAIP8 homolog CG4091/Sigmar led to abnormal salivary glands that have defects in the tubulin network and decreased autophagic flux [16]. The study also showed the interactions between Sigmar and several cytoskeletal proteins and the kinase Misshapen, which activate autophagy, both directly and indirectly [16]. Ha *et al*. [17] demonstrated that TNFAIP8 protein expression was induced by oxidative stress. Moreover, under stress conditions, TNFAIP8 interacts with F-Box and WD repeat domain containing 5 (FBXW5) to activate tubular sclerosis complex 2 (TSC2), a negative regulator of mTOR and a modulator of autophagy.

In this study, we investigated the role of TNFAIP8 in the regulation of cell-cycle-related proteins and autophagy in prostate and breast cancer cells. We demonstrated that alteration of TNFAIP8 in prostate cancer cells dysregulates cell-cycle-related genes (e.g., cyclin-dependent kinases [CDKs], cyclins, proliferating cell nuclear antigen [PCNA]), and autophagy markers and effectors (e.g., microtubule-associated protein 1A/1B-light chain 3 beta [LC3β] I/II, Beclin1, eukaryotic translation initiation factor 4E-binding protein 1 [4E-BP1], p62, sirtuin 1 [SIRT1]). In addition, by activation of autophagy, TNFAIP8 modulates the expression of neuroendocrine differentiation protein markers in prostate cancer cells. Ectopic expression of TNFAIP8 in cancer cells imparts resistance to TNF-α-induced apoptosis, which was associated with increased autophagy. These data suggest that TNFAIP8 promotes cell survival and drug resistance in prostate cancer cells by the induction of autophagy.

## RESULTS

### Analysis of TNFAIP8 expression in cancer cells

**Figure 1:**
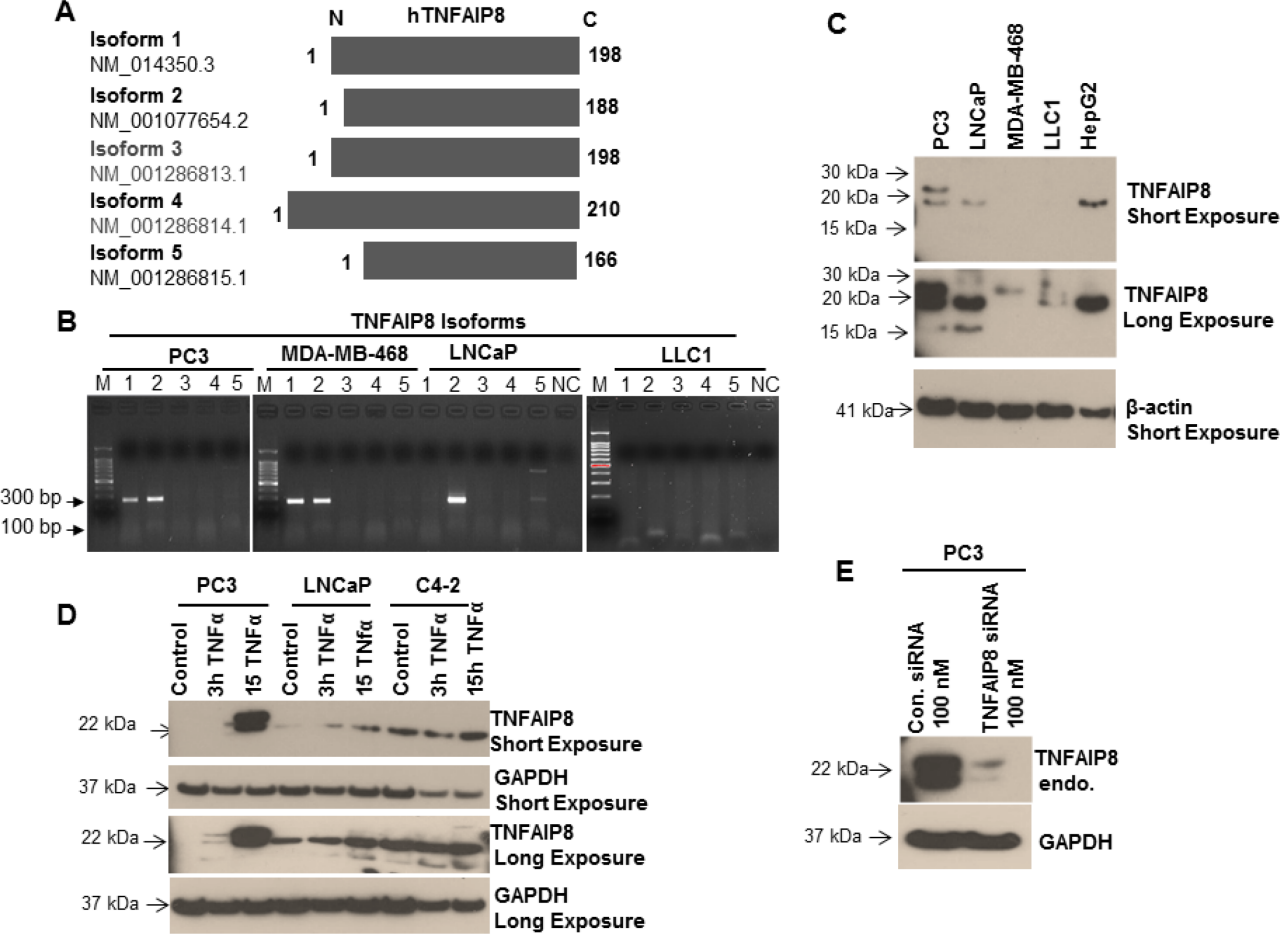
Analysis of the expression of TNFAIP8 isoforms in cancer cell lines (**A**) Amino acid sequence alignment of the five protein isoforms of human TNFAIP8. The N-terminal region is variable, whereas the C-terminal region is highly conserved. (**B**) Total RNA from PC3, MDA-MB-468, LNCaP, and LLC1 cells were isolated and the expression of the five TNFAIP8 isoforms was analyzed using RT-PCR. PCR products were electrophoresed on a 1.5% agarose gel and stained with ethidium bromide. NC, negative control (no cDNA); M, markers. (**C**) Expression of TNFAIP8 protein isoforms in various types of cancer cell lines was analyzed by immunoblotting. Cell extracts (50 μg) were electrophoresed using SDS-PAGE and immunoblotted with anti-TNFAIP8 and anti-β-actin antibodies. (**D**) PC3, LNCaP, and C4-2 cells were cultured for 24 h and treated with TNFα (10 ng/ml) for 3 h and 15 h. Cell extracts (50 μg) were immunoblotted with anti-TNFAIP8 and anti-GAPDH antibodies. (**E**) PC3 cells were transfected with control siRNA or TNFAIP8 siRNA (100 nM) for 30 h. Cell extracts (50 μg) were immunoblotted with anti-TNFAIP8 and anti-GAPDH antibodies. Endo–endogenous.

The expression pattern of the various TNFAIP8 isoforms in cancer cells has not been previously investigated. A previous amino-acid sequence alignment of the five reported TNFIP8 isoforms showed the N-terminal region had slight variations in their sequences and the C-terminal region was highly conserved (Figure 1A). We performed nucleotide sequence alignments of five reported TNFAIP8 transcript variants/isoforms (NCBI reference sequence: isoform 1- NM_014350.3, isoform 2- NM_001077654.2, isoform 3- NM_001286813.1, isoform 4- NM_001286814.1, and isoform 5- NM_001286815.1) and designed a unique forward primer for each isoform that annealed upstream of the translation initiation codon and a universal reverse primer from highly conserved coding region (Supplementary Figure 1A, left panel). The translation of isoforms 1 and 3 yields the same TNFAIP8 amino-acid sequence, but the 5ʹ upstream regions of these isoforms vary highly from one another. Isoform RNA expression was analyzed using RT-PCR in prostate (PC3, LNCaP), breast (MDA-MB-468), and mouse Lewis Lung Cancer 1 (LLC1) cells (Figure 1B). TNFAIP8 isoform 2 RNA was expressed in both prostate cancer cell lines and MDA-MB-468 cells but not in LLC1 cells. Isoform 1 RNA was observed in PC3 and MDA-MB-468 cells but not LNCaP or LLC1 cells. TNFAIP8 isoform 5 RNA was expressed at low levels in PC3 and LNCaP cells (Figure 1B). TNFAIP8 isoform RNA expression was also evaluated in THP-1 human monocytic leukemia cells and SH-SY5Y human neuroblastoma cells using RT-PCR (Supplementary Figure 1B, right panel). THP-1 cells expressed TNFAIP8 isoform 1, 2, and 5 RNA, whereas SH-SY5Y cells had no detectable expression of any TNFAIP8 isoform RNA. RNA expression of isoform 4 was not detected in any evaluated cell line. The expression of TNFAIP8 isoform RNA was validated by sequencing the gel bands.

The protein expression of TNFAIP8 isoforms was analyzed in PC3, LNCaP, MDA-MB-468, and LLC1 cells and human liver cancer cells (HepG2) by immunoblotting (Figure 1C). Consistent with the RT-PCR data, PC3 cells expressed high levels of isoform 1 and 2 proteins (molecular weight, approximately 23 kDa and 21 kDa, respectively) and low levels of isoform 5 protein (approximately 18 kDa). LNCaP cells expressed high levels of isoform 2 protein and low levels of isoform 5 protein, whereas LLC1 cells had no detectable expression of any TNFAIP8 isoform protein (Figure 1C). Interestingly, MDA-MB-468 cells only expressed isoform 1 protein, whereas HepG2 cells only expressed TNFAIP8 isoform 2 (Figure 1C). Because TNFAIP8 is a TNFα-inducible protein [12], we confirmed the expression and identity of TNFAIP8 isoforms by treating three prostate cancer cell lines (PC3, LNCaP, and C4-2) with TNFα and analyzing the induction of TNFAIP8 isoform proteins. Treatment with TNFα (10 ng/ml) induced a time-dependent increase in TNFAIP8 isoform protein expression in PC3, LNCaP, and C4-2 cells (Figure 1D). The TNFα-induced expression of TNFAIP8 isoforms in all three cell lines increased modestly after 15 h (Figure 1D).

To confirm the expression and specificity of TNFAIP8 isoforms, without induction of TNFAIP8 by TNFα, we knocked down TNFAIP8 protein expression in PC3 cells using siRNA (Figure 1E). Transfection of the TNFAIP8 siRNA reduced TNFAIP8 protein levels by 80–90% compared with control-siRNA-transfected cells. Collectively, these results clearly showed that TNFAIP8 isoforms expression is highly variable between different cancer cell lines.

### TNFAIP8 promotes prostate cancer cell survival

**Figure 2:**
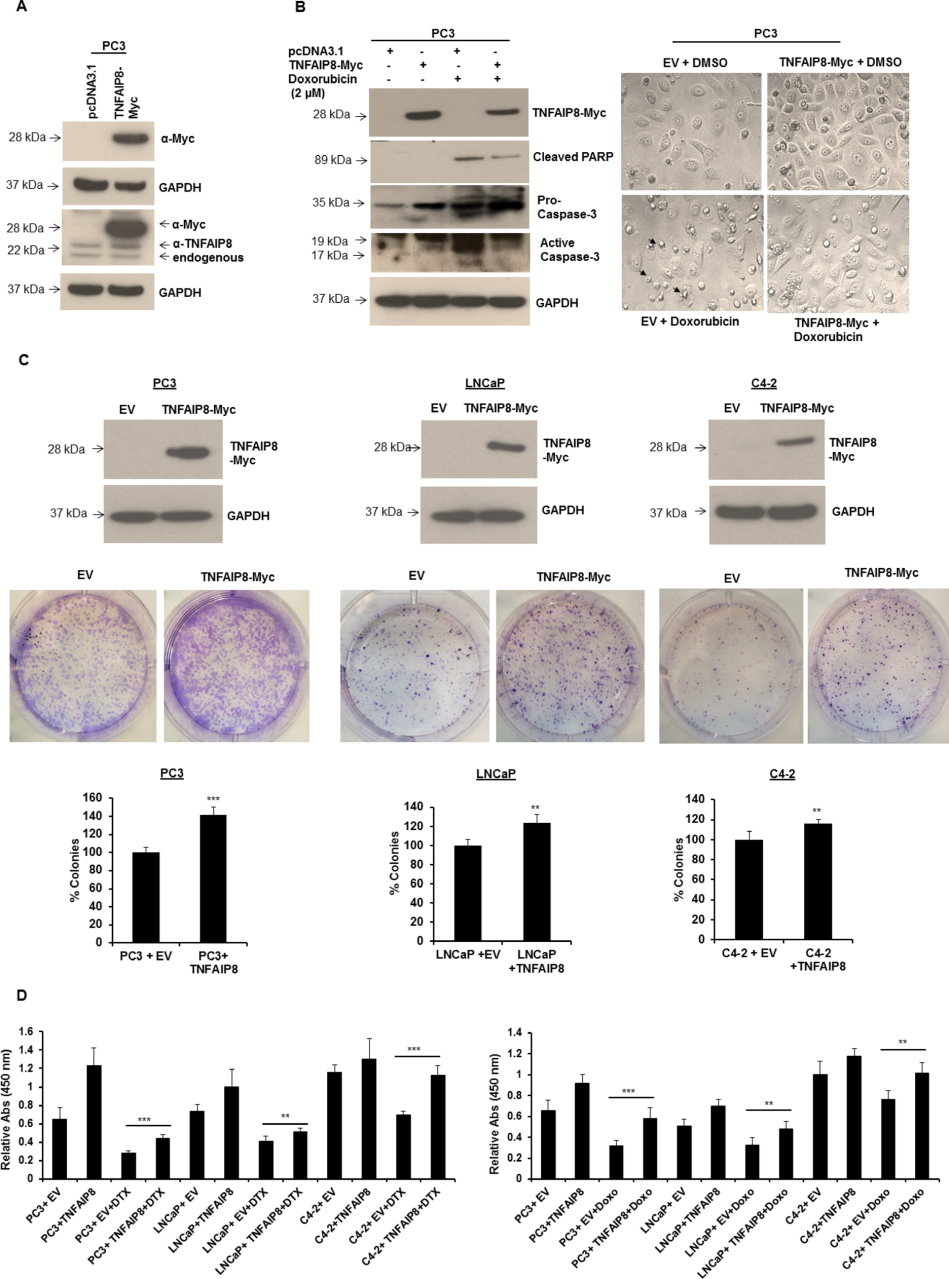
TNFAIP8 promotes cell colony formation and cell proliferation in prostate cancer cells (**A**) PC3 cells were transfected with empty vector (EV) or TNFAIP8-Myc plasmids for 24 h. Cell extracts (50 μg) were immunoblotted with anti-Myc, anti-TNFAIP8, and anti-GAPDH antibodies. (**B**) PC3 cells were transfected with empty vector or TNFAIP8-Myc plasmids for 24 h. Cells were treated with DMSO or 2 μM doxorubicin (Doxo) for 24 h. Cell extracts (50 μg) were immunoblotted with anti-Myc, anti-cleaved PARP, anti-caspase 3, and anti-GAPDH antibodies (left panel). PC3 cells (1 × 10^5^) were transfected with empty vector or TNFAIP8-Myc plasmid for 24 h. Cells were treated with DMSO or 2 μM doxorubicin for 24 h. Cells were imaged using a Nikon light microscope (40× objective; right panel) Arrows indicate the apoptotic cells. (**C**) PC3, LNCaP, and C4-2 cells were transfected with empty vector or TNFAIP8-Myc plasmid for 30 h. The expression of the TNFAIP8-Myc protein was analyzed by immunoblotting (upper panels). Transfected live cells (2000 cells/well) were re-plated in 6-well plates in triplicate and cultured at 37° C for 7 days. Cells were fixed, stained with crystal violet, counted, and plotted (middle and lower panels). (**D**) PC3, LNCaP, and C4-2 cells were transfected with the TNFAIP8-Myc plasmid or empty vector for 18 h and treated with DMSO or 0.5 nM of docetaxel (DTX) or 1 μM doxorubicin (Doxo) for 24 h. Cells were trypsinized and counted. Live cells (1 × 10^4^ cells/well) were cultured for 48 h, and cell proliferation was measured using a WST-1 assay. Data are expressed as the mean ± S.D. ^**^p < 0.01, ^***^p < 0.001, according to the two-tailed Student's *t*-test.

Because TNFAIP8 has been reported to have anti-apoptotic properties [6, 12], we confirmed the anti-apoptotic role of TNFAIP8 in prostate cancer cells. We transiently expressed TNFAIP8-Myc-tagged protein in PC3 cells and confirmed TNFAIP8-Myc protein expression by separately immunoblotting the lysates with anti-Myc-tagged and anti-TNFAIP8 antibodies (Figure 2A). TNFAIP8-Myc protein was expressed in PC3 cells transfected with the TNFAIP8-Myc plasmid and not those transfected with empty vector based on the dual detection of the same band in the immunoblots (Figure 2A). The blot with anti-TNFAIP8 antibody demonstrated that this antibody cross-reacts with TNFAIP8-Myc protein (referred to hereafter as TNFAIP8) in addition to endogenous TNFAIP8 isoforms (Figure 2A). To confirm the anti-apoptotic role of TNFAIP8 in prostate cancer cells, we transiently expressed TNFAIP8 protein in PC3 cells and treated them with doxorubicin. Cell lysates were then immunoblotted for the apoptotic markers for poly(ADP-ribose) polymerase (PARP) and caspase-3 (Figure 2B, left panel). TNFAIP8-transfected cells had higher levels of full-length procaspase-3 and lower levels of cleaved caspase-3 compared with empty-vector-transfected cells (Figure 2B, left panel). Doxorubicin treatment induced cleavage of caspase-3 and PARP proteins. However, the levels of cleaved caspase-3 and PARP decreased significantly following TNFAIP8 expression in doxorubicin-treated cells (Figure 2B, left panel). PC3 cell morphology and growth were evaluated following TNFAIP8 expression and under doxorubicin treatment conditions. TNFAIP8 expression promoted cell growth compared with empty-vector-transfected cells. Doxorubicin treatment induced cell apoptosis, but the number of apoptotic cells decreased significantly when TNFAIP8 was expressed in doxorubicin-treated cells (Figure 2B, right upper and lower panels).

We investigated the role of TNFAIP8 in cell survival using a cell colony formation assay and by measuring cell proliferation in three prostate cancer cell lines. PC3, LNCaP, and C4-2 cells were transfected with empty vector or TNFAIP8 plasmid for 24 h, and lysates were immunoblotted with anti-Myc antibody to confirm TNFAIP8 expression (Figure 2C, upper panel). Empty-vector or TNFAIP8-Myc transfected cells were transferred to 6-well plates and allowed to grow for 7 to 10 days. Following staining, colonies were counted and plotted (Figure 2C, middle and lower panels). The percentage colony formation of PC3, LNCaP, and C4-2 cells was significantly higher in TNFAIP8-transfected cells compared with empty-vector-transfected cells (Figure 2C, middle and lower panels). Then, PC3, LNCaP, and C4-2 cells were transfected with empty vector or TNFAIP8 plasmid for 18 h and treated with docetaxel or doxorubicin. The relative cell proliferation was measured using a WST-1 assay. TNFAIP8 overexpression decreased the sensitivity of all prostate cancer cell lines to docetaxel or doxorubicin. Thus, TNFAIP8 promotes cell proliferation (Figure 2D, left and right panels). Based, on this observation, it seems likely that TNFAIP8 modulates sensitivity to chemotherapies and may play a role in drug resistance. Collectively, these data suggest that TNFAIP8 promotes prostate cancer growth and increased drug resistance by inhibiting cellular apoptosis.

### TNFAIP8 dysregulates cell-cycle regulatory proteins

**Figure 3:**
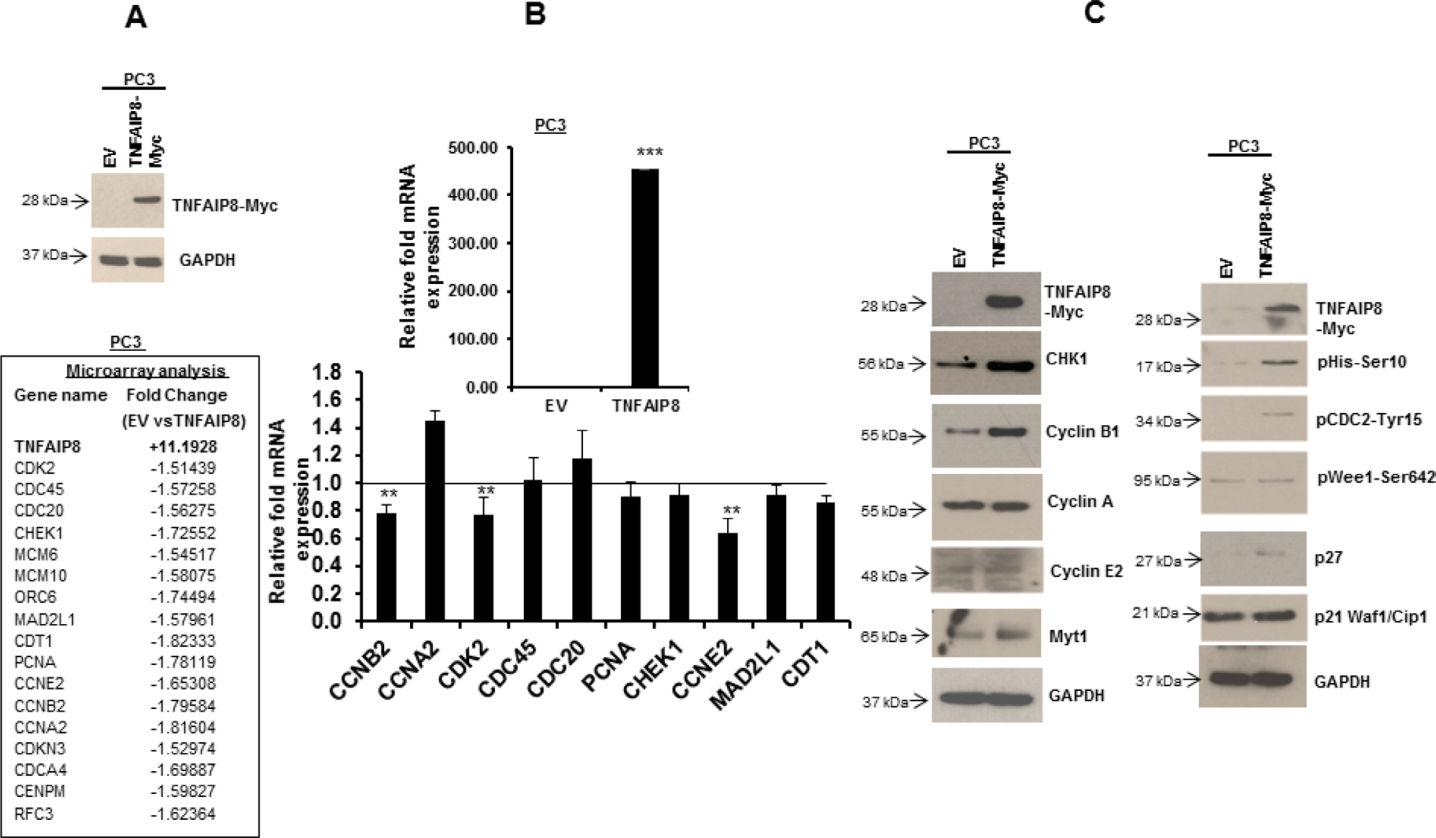
The effect of TNFAIP8 on the expression of cell-cycle-related proteins (**A**) PC3 cells were transfected with empty vector or TNFAIP8-Myc plasmid for 30 h. The expression of the TNFAIP8-Myc protein in cell extracts was analyzed by immunoblotting with anti-Myc and anti-GAPDH antibodies (upper panel). PC3 cells were transfected with empty vector or TNFAIP8-Myc plasmid for 30 h, and total RNA was isolated. The effect of TNFAIP8 gene expression on the expression of different cell-cycle-related genes was analyzed using microarray (lower panel). Fold-change represents the relative gene expression changes in empty-vector- verses TNFAIP8-Myc-transfected PC3 cells. (**B**) PC3 cells were transfected with empty vector or TNFAIP8-Myc plasmid for 30 h. The effect of expression of the TNFAIP8-Myc plasmid on the regulation of cell cycle-related genes was analyzed by RT/qPCR as described in materials and methods section. (**C**) PC3 cells were transfected with empty vector or TNFAIP8-Myc plasmid for 30 h. The effect of TNFAIP8 protein on the expression of cell cycle-related proteins was analyzed by immunoblotting of the cell extracts with antibodies against p21, p27, and phosphorylated Histone- H3, CDC2, and Wee1, and other indicated antibodies. Data are expressed as the mean ± S.D. ^**^p < 0.01, ^***^p < 0.001, according to the two-tailed Student's *t*-test.

TNFAIP8 has been implicated as an oncogenic molecule that regulates cancer cell progression in various human cancers [8–10]. To determine the molecular-level mechanisms underlying these functions, we expressed TNFAIP8 protein in PC3 cells (Figure 3A, upper panel) and performed microarray profiling. We assessed the effect of TNFAIP8 on global gene expression using a cut-off *p*-value < 0.01 and fold-change ≥ 1.5. TNFAIP8 expression was approximately 11-fold higher in TNFAIP8-transfected cells compared with empty-vector-transfected cells. Microarray analysis revealed that several genes associated with cell cycle (e.g., checkpoint kinase 1 [CHEK1], CDK2, cell division cycle (CDC20, CDC45, CDCA4) and DNA replication or silencing (e.g., chromatin licensing and DNA replication factor 1 [CDT1], origin recognition complex subunit 6 [ORC6], minichromosome maintenance 6 [MCM6], MCM10, PCNA) were downregulated (1.5 to 1.8-fold) in TNFAIP8-Myc plasmid transfected cells compared with empty vector-transfected PC3 cells (Figure 3A, lower panel). To validate the microarray data, we performed RT/qPCR. RT/qPCR data demonstrated that the ectopic expression of TNFAIP8 gene downregulates the expression of several cell cycle-related gene transcripts including CCNB2, CDK2, CCNE2, and others (Figure 3B, upper and lower panels). In addition, we immunoblotted lysates from TNFAIP8-transfected cells and analyzed the expression of key cell-cycle regulatory proteins such as cyclins, Myt1, p21 and p27. When comparing TNFAIP8- and empty-vector-transfected PC3 cells, TNFAIP8 overexpression upregulated CHEK1, Cyclin B, and myelin transcription factor 1 (MYT1) proteins and induced phosphorylation of Histone-H3 at Ser10, CDC2 at Tyr15, and WEE1 G2 checkpoint kinase (Wee1) at Ser642 (Figure 3C, left panel). However, no changes in CDK inhibitors, such as p21 and p27, were observed (Figure 3C, right panel). These data suggest that TNFAIP8 dysregulates cell-cycle-related proteins and possibly modulates cell-cycle progression.

To explore the role of TNFAIP8 in cell-cycle regulation, we expressed TNFAIP8 in PC3 and LNCaP cells and monitored cell-cycle progression using flow cytometry (Supplementary Figure 2). Cells expressing TNFAIP8 did not display any cell-cycle changes or cell-cycle arrest. The data suggest that the regulation of cell-cycle regulatory proteins by TNFAIP8 does not seem to directly affect the cell-cycle progression.

### TNFAIP8 induces autophagy

**Figure 4:**
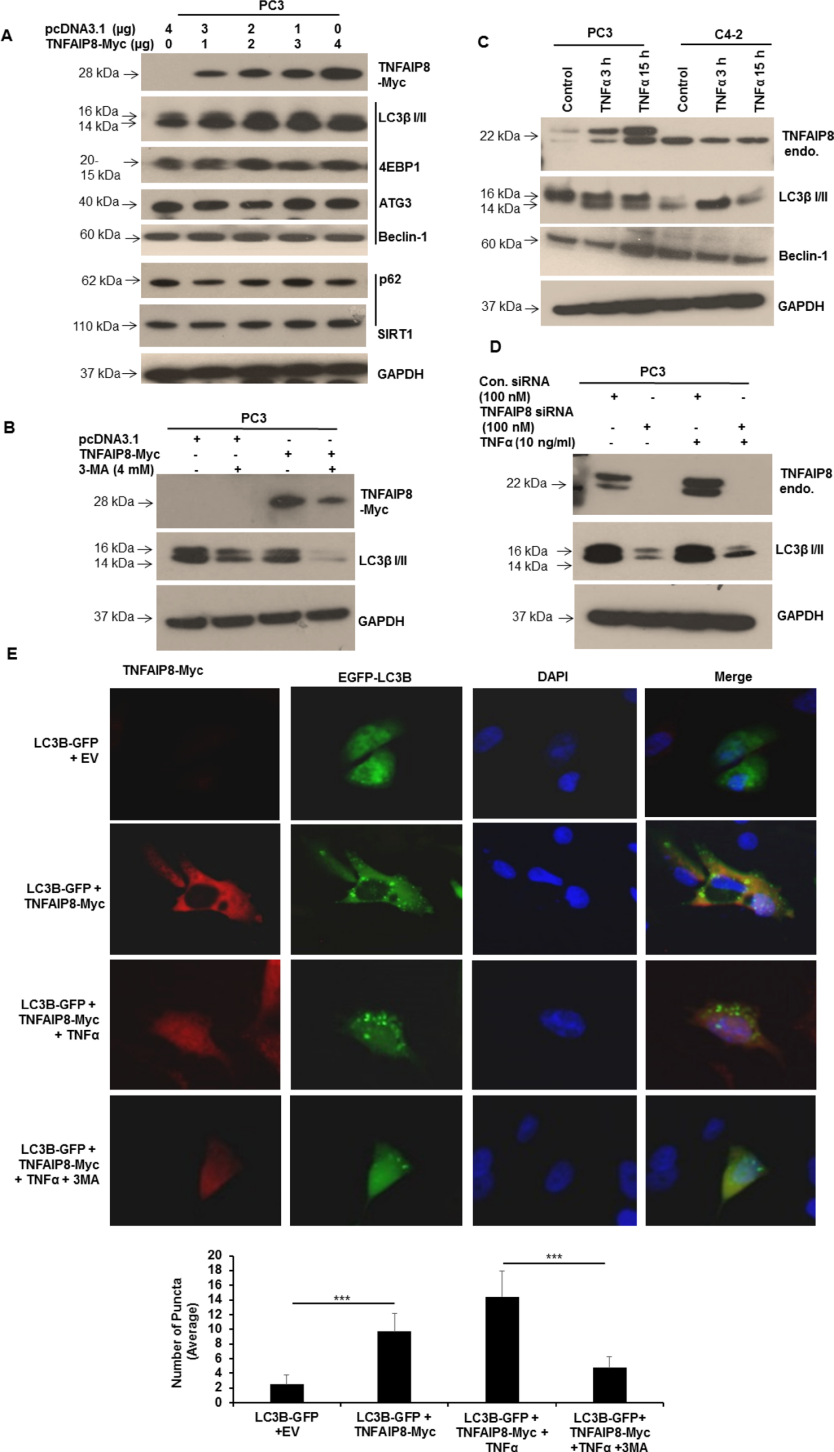
TNFAIP8 induces autophagy in PC3 cells (**A**) PC3 cells were transfected with empty vector or increasing amounts of TNFAIP8-Myc plasmid for 30 h. Cell extracts (50 μg) were electrophoresed using SDS-PAGE and immunoblotted with anti-Myc, anti-LC3β I/II, anti-4E-BP1, anti-ATG3, anti-Beclin 1, anti-p62, anti-SIRT1, and anti-GAPDH antibodies. (**B**) PC3 cells were transfected with empty vector or TNFAIP8-Myc plasmid and treated with 3-methyladenine (4 mM) for 24 h. Cell extracts (50 μg) were immunoblotted with anti-Myc, anti-LC3β I/II, and anti-GAPDH antibodies. (**C**) PC3 and C4-2 cells were cultured for 24 h and treated with TNFα (10 ng/ml) for 3 h and 15 h. Cell extracts (50 μg) were immunoblotted with anti-TNFAIP8, anti-LC3β I/II, anti-Beclin-1, and anti-GAPDH antibodies. Endo–endogenous. 3-MA – 3-methyladenine. (**D**) PC3 cells were transfected with control siRNA or TNFAIP8 siRNA (100 nM) and treated with TNFα (10 ng/ml) for 24 h as indicated. Cell extracts (50 μg) were immunoblotted with anti-TNFAIP8, anti-LC3β I/II and anti-GAPDH antibodies. Endo–endogenous. (**E**) PC3 cells were grown on coverslips in 6-well plates for 24 h and co-transfected with 2 μg of empty vector and GFP-tagged-LC3B plasmid or TNFAIP8-Myc plasmid and GFP-tagged LC3B plasmid. Cells were treated with 10 ng/ml TNFα and 4 mM 3-methyladenine for 24 h as indicated. TNFAIP8 was labeled using anti-Myc antibody and an Alexa-Fluor-568-conjugated secondary antibody. Nuclei were stained with DAPI. Cells were imaged using an OlympusBX60 fluorescent microscope (40× objective) (upper panels). The number of GFP-LC3B-related puncta formed in cells (*n* = 10) was counted and plotted (lower panels). Data are expressed as the mean ± S.D. ^***^p < 0.001, according to the two-tailed Student's *t*-test.

Autophagy plays an important role both in cell survival and apoptosis, and the interplay between cell-cycle proteins and autophagy has been well-documented [18, 19]. Because TNFAIP8 is an oncogenic molecule that dysregulates the expression of multiple cell-cycle-related proteins and promotes cancer cell growth (Figures 3, 2), and therefore we hypothesized that TNFAIP8 may promote cancer cell growth by inducing autophagy. We transfected PC3 cells with empty vector or increasing amounts of TNFAIP8 plasmid and analyzed the effect of TNFAIP8 expression on autophagy markers by Western blotting (Figure 4A). TNFAIP8 expression in PC3 cells increased the expression of LC3β I/II, 4E-BP1, autophagy-related protein 3 (ATG3), and Beclin 1 in a dose-dependent manner, indicating that TNFAIP8 induces autophagy (Figure 4A). TNFAIP8 expression also stabilized p62, a protein that recognizes and facilitates the degradation of toxic biomolecules through autophagy [20]. Further, TNFAIP8 expression increased SIRT1 levels (Figure 4A). SIRT1 is a NAD-dependent deacetylase that regulates the nucleocytoplasmic translocation of LC3β I/II through deacetylation. This acetylation-deacetylation cycle helps to regulate autophagy [21, 22]. SIRT1 upregulation by TNFAIP8 suggests that LC3 stability and localization play a role in TNFAIP8-induced autophagy. Next, we studied the autophagy-inducing effects of TNFAIP8 using the autophagy inhibitor 3-methyladenine. PC3 cells were transfected with TNFAIP8 plasmid and treated with 3-methyladenine for 24 h. Then, we analyzed LC3β I/II expression by immunoblotting. TNFAIP8 increased the expression of LC3β I/II, whereas treatment of TNFAIP8-transfected cells with 3-methyladenine reduced LC3β I/II expression (Figure 4B). These data suggest that TNFAIP8 can induce cellular autophagy in PC3 cells.

### TNFAIP8 is essential for TNFα-induced autophagy

TNFα has been shown to increase autophagy in multiple cells [23–25]. We confirmed this observation by treating PC3 and C4-2 cells with TNFα. LC3β I/II expression was enhanced in both cell lines following treatment (Figure 4C). Because TNFAIP8 is a TNFα-inducible protein that helps protect cells from TNFα-induced cell death [4], we hypothesized that TNFAIP8 plays a role in TNFα-induced autophagy. First, we confirmed treatment with TNFα induced TNFAIP8 expression and autophagy, as indicated by LC3β I/II. Then, we knocked down TNFAIP8 expression using siRNA, treated cells with TNFα to induce autophagy, and assessed autophagy by immunoblotting. TNFAIP8 knockdown significantly decreased LC3β I/II expression compared that of control-siRNA-transfected cells (Figure 4D). TNFAIP8 knockdown inhibited TNFα-induced autophagy, suggesting a critical role for TNFAIP8 in this process.

Next, we studied TNFα-induced autophagy using immunofluorescence. When GFP-tagged LC3β plasmid was co-transfected with TNFAIP8 plasmid and when GFP-tagged-LC3β-transfected cells were treated with TNFα, GFP-LC3β formed puncta (Figure 4E, upper and lower panels). Treatment with 3-methyladenine reduced TNFAIP8-mediated LC3β puncta formation suggesting that TNFAIP8 is involved in modulating cellular autophagy.

### TNFAIP8 expression is associated with autophagic flux in normal and breast cancer cells

In order to establish the broader role of TNFAIP8 in autophagy modulation, we analyzed the expression of TNFAIP8 and LC3β I/II in normal and breast cancer cells by immunoblotting. Breast cancer cell lines included MCF-7 MCF7-RAS, MCF7-Adriamycin resistant (ADR); normal immortalized breast cell lines included MCF10A, MCF10A-Neo, MCF10A-RAS/ErbB2 and MCF10A-TGFα. MCF7-RAS, MCF10A-RAS/ErbB2, and MCF10A-TGFα cells stably express RAS, ErbB2, and TGFα, respectively [26–30]. TNFAIP8 expression was 2–3-fold higher in MCF10A-RAS/ErbB2 and MCF10A-TGFα cells than in MCF10A cells or empty-vector-transfected MCF10A cells (Supplementary Figure 3A, lanes 4–7). The higher TNFAIP8 expression in MCF10A-RAS/ErbB2 and MCF10A-TGFα cells induced the expression of LC3β II isoforms compared with MCF10A cells or empty-vector-transfected MCF10A cells (Supplementary Figure 3A, lanes 4–7). Further, we observed higher autophagic flux, as indicated by LC3β I/II, in MCF-10A cell lines expressing higher TNFAIP8 levels, suggesting TNFAIP8 is associated with autophagic flux. Interestingly, MCF-7, MCF7-RAS, and MCF7-ADR cells showed lower expression levels of both TNFAIP8 and LC3β I/II compared with MCF10A cells (Supplementary Figure 3A, lanes 1–4). Collectively, these data suggest that TNFAIP8 expression may be associated with higher autophagic flux in MCF10A breast epithelial cells compared with MCF-7 breast cancer cells.

Next, we selected the MCF-7 cells, which expressed the lowest levels of TNFAIP8 and evaluated the effect of ectopic TNFAIP8 expression with serum starvation on LC3β I/II expression by immunoblotting. Overexpression of TNFAIP8 in MCF-7 cells induced autophagy in both serum-starved and complete-media-grown cells at 24 h and 48 h post-transfection (Supplementary Figure 3B, left & right panels). Collectively our results suggest that TNFAIP8 increases cellular autophagy in breast cancer cells.

### TNFAIP8 interacts with ATG3, induces autophagy, and reduces cellular apoptosis

**Figure 5:**
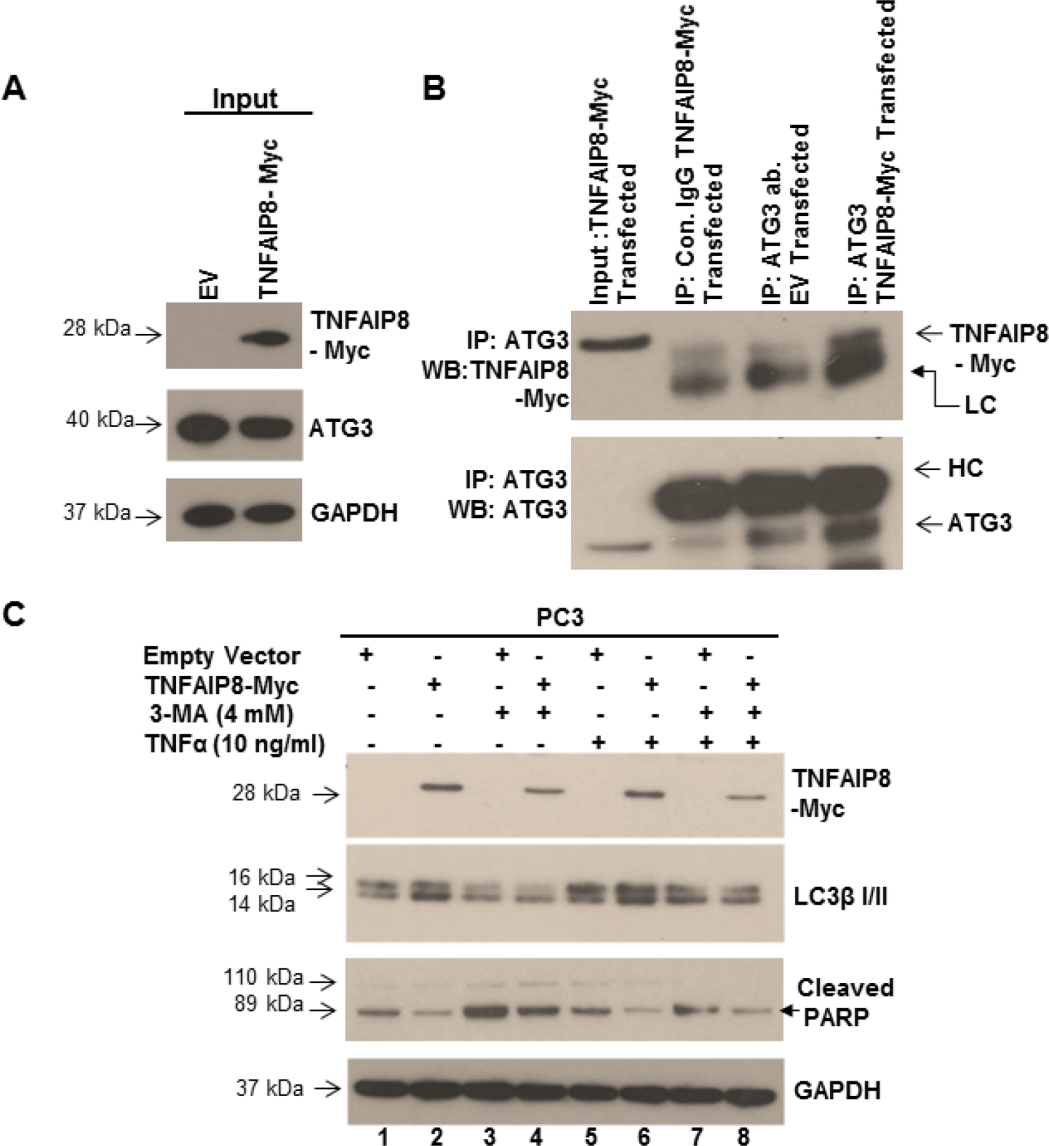
TNFAIP8 interacts with ATG3, induces autophagy, and inhibits apoptosis (**A**) PC3 cell extracts (50 μg) from empty vector- or TNFAIP8-Myc-transfected cells were immunoblotted with anti-Myc, anti-ATG3, and anti-GAPDH antibodies (input panel). (**B**) Cell lysates (1 mg) from empty vector- or TNFAIP8-Myc-transfected PC3 cells were immunoprecipitated with control IgG or anti-ATG3 antibody and immunoblotted with anti-TNFAIP8-Myc and anti-ATG3 antibody. LC- light-chain IgG, HC- heavy-chain IgG. (**C**) PC3 cells were transfected with empty vector or TNFAIP8-Myc plasmid and treated with 3-methyladenine (4 mM) and TNFα (10 ng/ml) for 24 h as indicated. Cell extracts (50 μg) were immunoblotted with anti-Myc, anti-TNFAIP8, anti-LC3β I/II, anti-cleaved PARP, and anti-GAPDH antibodies.

TNFAIP8 has been reported to interact with several cytoskeletal proteins, such as Act42 and alpha TUB4B in Drosophila. These proteins participate in the autophagy process, either directly or indirectly [16, 31]. A high-throughput interactome study by Kristensen *et al*. [32] demonstrated that TNFAIP8 interacts with ATG3. Because our data showed that TNFAIP8 modulated autophagy markers, we hypothesized that TNFAIP8 may interact with ATG3 or other autophagy-related proteins in prostate cancer cells. To determine if TNFAIP8 interacts with ATG3, we transfected PC3 cells with TNFAIP8 or empty vector (Figure 5A, input panel), immunoprecipitated cell lysates with anti-ATG3 antibody or control antibody, and analyzed interactions by immunoblotting with anti-TNFAIP8 or anti-ATG3 antibody as indicated (Figure 5B). ATG3 co-immunoprecipitated with TNFAIP8 in TNFAIP8-transfected cells but not empty-vector-transfected cells (Figure 5B), suggesting that TNFAIP8 increases autophagy through an interaction with ATG3 or one of the components of the autophagosome.

Next, we investigated the biological significance of TNFAIP8-induced autophagy with respect to apoptosis. PC3 cells were transfected with the TNFAIP8-Myc plasmid or empty vector and cell were treated with vehicle or TNFα to induce apoptosis in the presence or absence of the autophagy inhibitor 3-methyladenine. Following treatments, autophagy was detected by immunoblotting. Compared with empty-vector-transfected cells, TNFAIP8 overexpression in PC3 cells increased LC3β I/II expression and decreased cleaved PARP levels, indicating autophagy initiation and apoptosis inhibition, respectively (Figure 5C, lane 1&2). Treatment with 3-methyladenine reduced LC3β I/II expression and increased cleaved PARP levels significantly (Figure 5C, lane 2 vs 4) in TNFAIP8-transfected cells compared with untreated TNFAIP8-transfected cells. The combination of TNFAIP8 overexpression and TNFα treatment increased LC3β I/II expression and reduced cleaved PARP levels significantly compared with empty-vector-transfected TNFα-treated cells and with empty-vector-transfected TNFα-and 3-methyladenine-treated cells (Figure 5C, lanes 5–8). These data clearly showed that TNFAIP8 expression inhibits TNFα-mediated apoptosis by increasing autophagy in PC3 cells.

### TNFAIP8 is involved in the modulation of neuroendocrine differentiation markers

**Figure 6:**
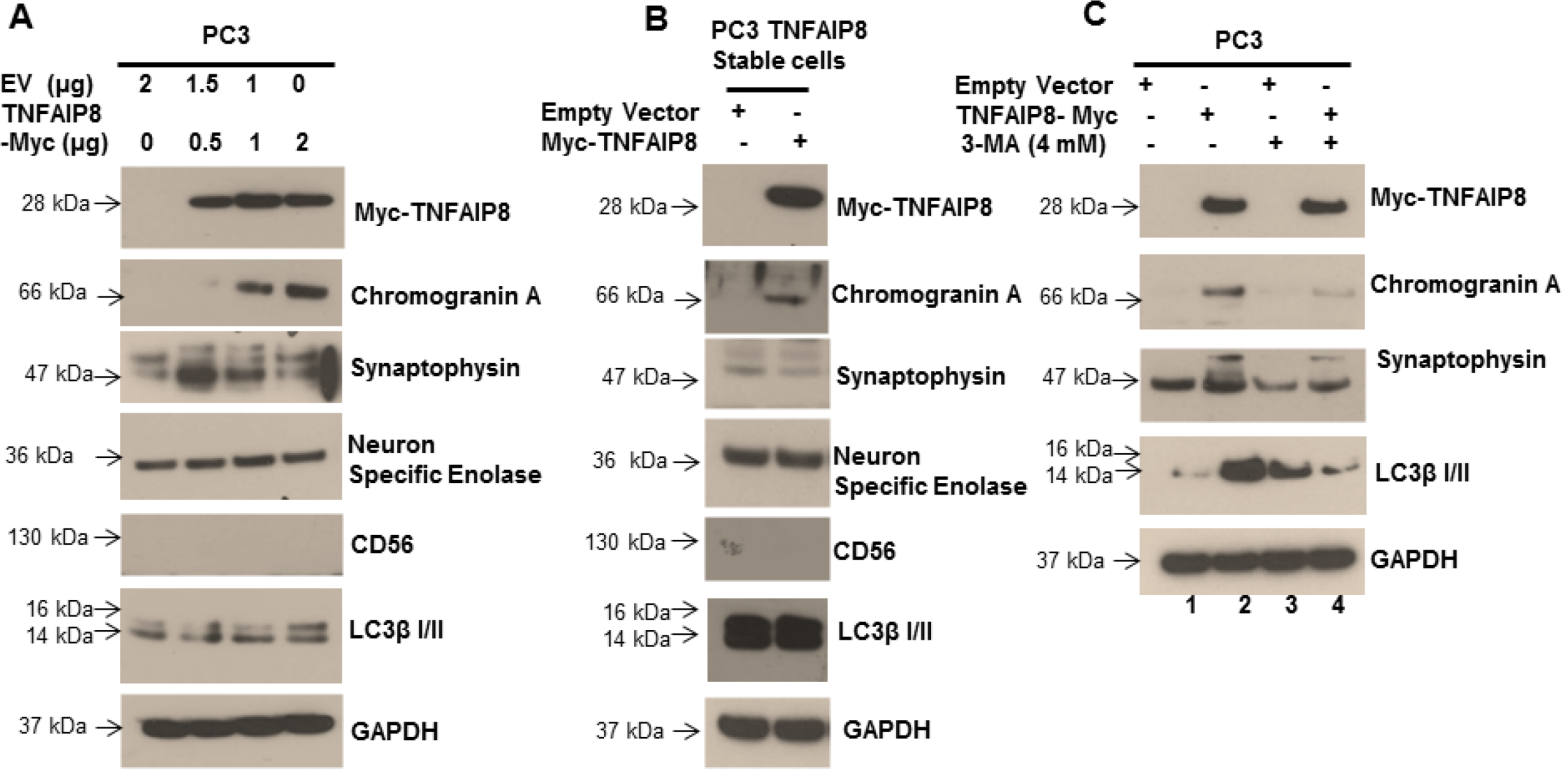
TNFAIP8 increases the expression of NED biomarkers in prostate cancer PC3 cells (**A**) PC3 cells were transfected with empty vector or increasing amounts of TNFAIP8-Myc plasmid for 30 h. The effect of TNFAIP8 protein expression on NED biomarkers, such as chromogranin A, synaptophysin, NSE, and NCAM-1/CD56, as well as the autophagy marker LC3β I/II was analyzed by immunoblotting. (**B**) Effect of stable TNFAIP8 expression on NED markers in PC3 cells. Cell extracts from PC3 cells stably expressing TNFAIP8 were analyzed by immunoblotting with the indicated antibodies. (**C**) PC3 cells were transfected with empty vector or TNFAIP8-Myc plasmid and treated with 3-methyladenine for 24 h. The effect of TNFAIP8 expression, with and without 3-methyladenine, on the expression of NED and autophagy markers, was analyzed by immunoblotting.

Previous studies showed that activation of the autophagy signaling pathway contributes to neuroendocrine differentiation in prostate cancer cells [33, 34], and our results demonstrated that TNFAIP8-induced autophagy was not limited to prostate cancer cells. To determine if TNFAIP8 regulates neuroendocrine differentiation in prostate cancer cells by the activation autophagy, we transfected PC3 cells with varying concentrations of TNFAIP8 plasmid and evaluated the expression of neuroendocrine differentiation (NED) biomarkers, such as synaptophysin, neuron-specific enolase (NSE), chromogranin A, and NCAM-1/CD56 (Figure 6A). Expression of TNFAIP8 in PC3 cells increased the expression of LC3β I/II and the NED biomarkers chromogranin A and synaptophysin in a dose-dependent manner but did not affect the expression of NSE (Figure 6A). We also tested the expression of NED biomarkers in PC3 cells that stably expressed TNFAIP8 (Figure 6B). The stable expression of TNFAIP8 in PC3 cells increased the expression of chromogranin A compared with normal PC3 cells but did not affect the expression of synaptophysin or NSE. The expression of NCAM-1/CD56 was not detected in either cell line (Figure 6A and 6B). We also examined the effects of TNFAIP8 and 3-methyladenine on the regulation of chromogranin A and synaptophysin in PC3 cells. TNFAIP8 expression induced the expression of chromogranin A and synaptophysin as well as LC3β I/II in TNFAIP8-transfected cells compared with empty-vector-transfected cells (Figure 6C, lane 1&2). Treatment with 3-methyladenine, both with and without TNFAIP8 expression, reduced the expression of chromogranin A, synaptophysin, and LC3β compared with untreated PC3 cells (Figure 6C, lane 3&4), suggesting that TNFAIP8 increases NED by the activation of autophagy in PC3 cells (Figure 6C). Collectively, the data suggest that the activation of cellular autophagy by TNFAIP8 might be involved in the neuroendocrine differentiation in prostate cancer cells.

## DISCUSSION

TNFAIP8 is an anti-apoptotic protein that helps inhibit TNFα mediated cellular apoptosis. The TNFAIP8 expression is induced following nuclear factor-κB (NF-κB) activation and is controlled by various other factors. Promoter analysis of *TNFAIP8* revealed potential binding sites for transcription factors, such as hypoxia-inducible factor (HIF), nuclear receptor subfamily 2 group F member 1 (NR2F1), and androgen receptor [12, 35]. TNFAIP8 expression increases significantly in various cancer cell lines, leading to cancer progression and poor prognosis [8–10, 12]. Thus far, four TNFAIP8 protein isoforms have been reported; however, the expression levels and unique functions of each isoform are still unknown. Interestingly, all four isoforms of TNFAIP8 shared more than 90% of amino-acid sequence homology with highly conserved C-terminal regions. In the current study, we analyzed the expression profile of TNFAIP8 isoforms in prostate, breast, and liver cancer cell lines and found that isoform 2 is the predominantly expressed isoform in prostate and liver cancer cells. RT-PCR and immunoblotting data suggested that other TNFAIP8 isoforms are also expressed in various cancer cell lines. However, the individual role of TNFAIP8 isoforms in cancer cell biology needs to be further investigated.

The TNFAIP8 protein family is involved in various functions in human diseases, including cancer [5, 6, 11]. Several studies showed that TNFAIP8 plays a role in the cellular anti-apoptotic process and promotes cellular growth and proliferation in various cancers [6, 8–11]. However, the molecular mechanism underlying how TNFAIP8 promotes cell survival is still unknown. We investigated the role of TNFAIP8 in modulating the expression of cell-cycle-related proteins, autophagy biomarkers, and drug resistance in prostate and breast cancer cell lines. The data suggested that overexpression of TNFAIP8 reduced the expression of cell-cycle-related several proteins, such as cyclins and CDKs. However, no substantial TNFAIP8-mediated cell-cycle arrest was observed. Recent studies showed that dysregulation of cell-cycle-related protein modulates cellular autophagy and there is a direct interplay between cell-cycle-related proteins and autophagy modulators [18, 19]. Because autophagy plays an important role in both tumor development and cancer cell survival [36], we investigated whether TNFAIP8 is involved in cellular autophagy via dysregulation of cell-cycle-related proteins. Recently, a TNFAIP8-related proteomic analysis showed that TNFAIP8 interacts with several cytoskeletal proteins, namely Act42 and alpha Tub84B in Drosophila. These cytoskeletal proteins participate in initiating cellular autophagy, directly or indirectly [16, 31]. Using high-throughput analysis of changes in the interactome, earlier studies showed that TNFAIP8 directly interacts with ATG3 [32], indicating TNFAIP8 may participate in the initiation of autophagy. Our data support this hypothesis; moreover, we showed that TNFAIP8 interacts with ATG3 and increases the expression of autophagy markers and effectors, such as LC3β I/II, Beclin1, and 4E-BP1 in PC3 cells. TNAIP8 also stabilized p62 and SIRT1, which are directly involved in controlling cellular autophagy. Knockdown of TNFAIP8 reduced the expression of LC3β I/II in breast cancer MCF7 cells (data not shown) and prostate cancer PC3 cells, which reinforces the role of TNFAIP8 in LC3β I/II regulation and autophagy.

**Figure 7:**
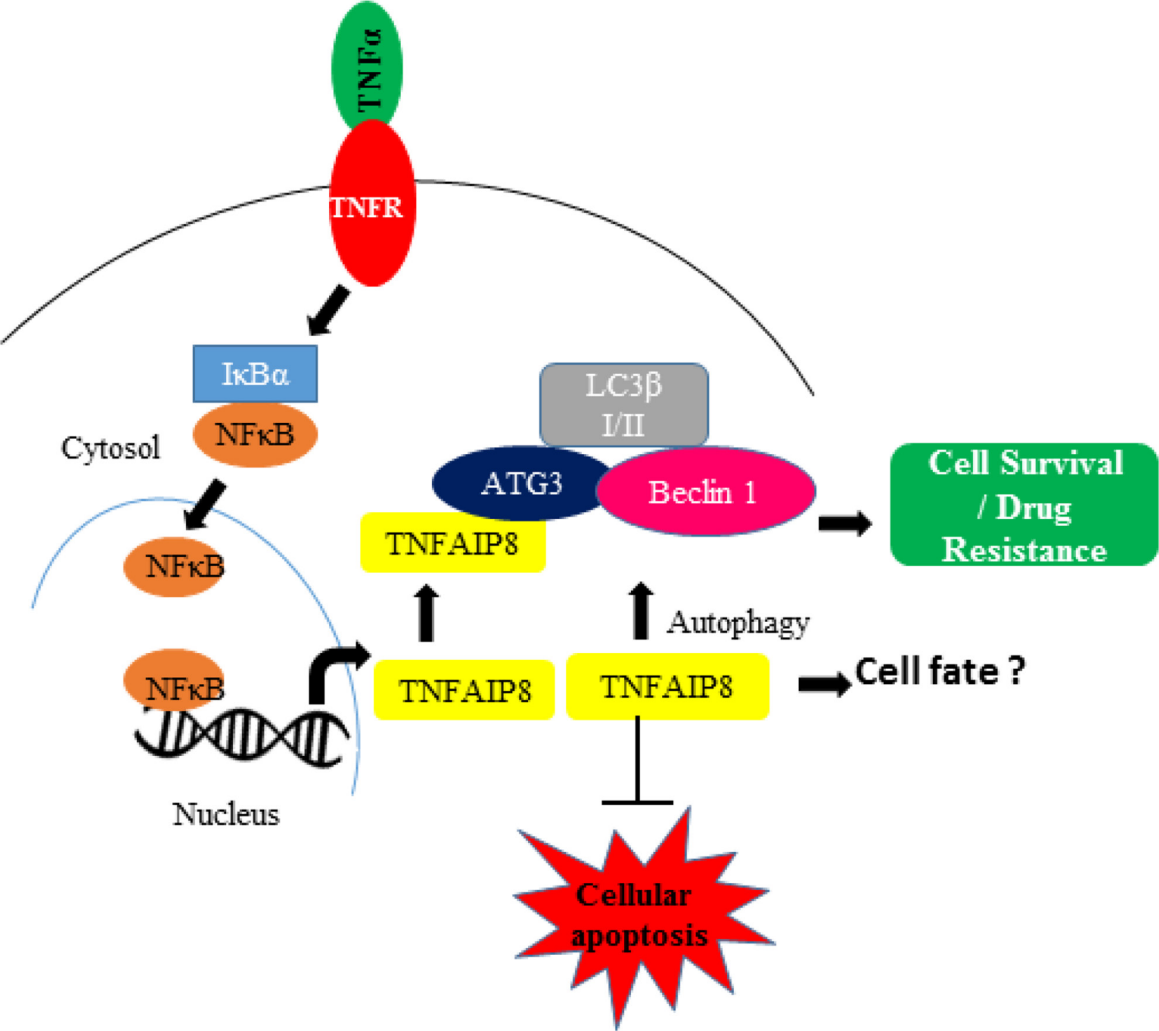
The involvement of the TNFAIP8 protein in autophagy The model represents the expression and involvement of TNFAIP8 in autophagy formation. The model also indicates the important role of TNFAIP8 in determining cell fate during TNFα signaling.

Autophagy is a vital self-degradation process that eliminates damaged organelles and misfolded or aggregated proteins through the lysosomal degradation pathway [36–38]. Autophagy maintains normal cell homeostasis, and autophagic deregulation is associated with several pathological processes, including several cancers. Several reports have suggested that autophagy can affect chemotherapeutic and immunotherapeutic response in cancer cells [39, 40]. Importantly, autophagy both positively and negatively regulates cell death, and recently, it has been shown that the response to death receptor activation is based on basal autophagy levels. In most tumor cells, TNFAIP8 has been reported to have a protective effect; however, TNFAIP8 promotes glucocorticoid-induced apoptosis in thymocytes [41]. Our data suggest that treatment with TNFα induced the expression of TNFAIP8 and the autophagy marker LC3β I/II. This induction led to inactivation of cellular apoptosis by decreasing the cleavage of apoptotic PARP and caspase-3, subsequently promoting cell survival (Figure 7). We also showed that TNFAIP8 increased resistance to the anticancer drugs, docetaxel, and doxorubicin, and promoted cell proliferation and cell growth in prostate cancer cells through the inactivation of cellular apoptosis and the induction of cellular autophagy. Previous studies showed that activation of cellular autophagy contributes to NED in prostate cancer cells [33, 34]. Here, we demonstrated that TNFAIP8 expression induced expression of the NED biomarkers chromogranin A and synaptophysin in prostate cancer cells; however, the exact mechanism by which TNFAIP8 induces NED in prostate cancer cells remains unclear.

In conclusion, this study provides first-time evidence that TNFAIP8 protein participates in the initiation of cellular autophagy and modulates cell survival in prostate cancer cells. The role of TNFAIP8 in cell death or survival appears to depend on the cellular context. TNFα is a cytokine involved in systemic cellular inflammation, leading to cell death. Future studies should further investigate whether the level of TNFAIP8 expression induced by TNFα determines cell fate by modulating autophagy-like events and if TNFAIP8 could act as a critical gatekeeper between cell survival and cell death.

## MATERIALS AND METHODS

### Cell culture

Cancer cell lines were obtained from Georgetown University Lombardi Comprehensive Cancer Center cell culture repository. Prostate cancer (LNCaP, PC3, C4-2) and breast cancer (MCF7 and MDA-MB-468) and liver cancer HepG2 cells were grown in appropriate medium (RPMI or DMEM from Invitrogen) containing 5–10% Fetal Bovine Serum (FBS, Access Biologicals, Vista, CA, USA), 2 mM glutamine, and 25 μg/ml gentamicin (Invitrogen) and incubated at 37° C with 5% CO_2_. MCF-7, MCF7-RAS, MCF7-ADR, MCF10A, MCF10A-Neo, MCF10A-RAS/ErbB2 and MCF10A-TGFα cells were also obtained from Georgetown University Lombardi Comprehensive Cancer Center cell culture repository and maintained as described previously [26–30]. Mouse Lewis lung carcinoma-1 (LLC-1) cells were obtained from Dr. Micheler Richardson's laboratory, and human monocytic leukemia THP-1 cells and human neuroblastoma SH-SY5Y cells from Dr. K. Sean Kimbro's laboratory (BBRI-North Carolina Central University, NC). We also generated a stable PC3 cell line which expressed TNFAIP8-Myc-tagged protein. For this, PC3 cells were transfected with empty vector or TNFAIP8-Myc-tagged plasmid for 30 h. Cells were trypsinized, re-plated, and treated with 600 μg/ml G418 for 2 weeks. Antibiotic-containing medium was replaced every 3–4 days; cell colonies were selected, expanded, and maintained in presence of G418. TNFAIP8-Myc-tagged protein expression was confirmed by immunoblotting. All cell lines were grown at least 24 h and used for experiments once they reached 70–80% confluence.

### Western blot analysis

Immunoblotting analysis was performed following standard procedures. Cells were lysed in cell lysis buffer (Cell Signaling, Danvers, MA, USA) containing protease inhibitor (Roche, Indianapolis, IN, USA). After centrifugation at 10,000 rpm for 15 min, the protein concentrations from the supernatants were estimated using a Bio-Rad protein assay (Bio-Rad, Hercules, CA, USA). The proteins (30–50 μg) were separated on a NuPAGE 4–12% Bis-Tris-SDS gel (Invitrogen) and transferred onto a PVDF membrane (Immobilon-P, Millipore, Billerica, MA, USA). Membranes were blocked in 1× blocking buffer (Sigma-Aldrich, St. Louis, MO, USA) for 1 h and incubated in primary antibody overnight at 4° C. We used anti-TNFAIP8 antibody from Proteintech Group (Cat. #15790-1-AP). Anti-CHK1, anti-cyclin B1, anti-cyclin A, anti-cyclin E2, anti-phospho-histone H3 (Ser10), anti-phospho-cdc2 (Tyr15), anti-phospho-Wee1(Ser642), anti-p21 (Waf1/Cip1), anti-Myt1, anti-LC3β I/II, anti-4E-BP1, anti-SIRT1, anti-cleaved PARP, anti-caspase-3, anti-Myc tag, and anti-GAPDH antibodies were obtained from Cell Signaling Technology (Danvers, MA, USA). Anti-p27 and anti-Beclin1 were obtained from Santa Cruz Biotechnology (Dallas, TX, USA), anti-p62 from BD Bioscience (San Jose, CA, USA), and anti-ATG3 and anti-β-actin from Sigma. Prostate-cancer-specific NED-biomarker antibodies, such as anti-synaptophysin, anti-NSE, anti-chromogranin A, and anti-NCAM-1/CD56 were purchased from Novus Biologicals (Littleton, CO, USA). All antibodies were used according to the manufacturer›s suggestions. After washing the membranes three times with Tris-buffered saline with 0.1% Tween 20 (TBST), the membranes were incubated in appropriate secondary antibody (1:10000 dilution) (Jackson ImmunoResearch, PA) for 1 h at room temperature, and immunoreactive bands were visualized using ECL chemiluminescence detection (Signagen Laboratories, Rockville, MD, USA).

### Immunoprecipitation

Cell extracts (1 mg) from empty-vector transfected or TNFAIP8-Myc-transfected PC3 cells were equilibrated in radioimmunoprecipitation assay buffer (RIPA; 50 mm Tris pH 8.0, 150 mM NaCl, 0.2 mM EDTA, 1% Nonidet P-40, and 1 mM phenylmethylsulfonyl fluoride, supplemented with protease inhibitor [Roche Applied Science]). The lysates were cleared with protein AG plus-agarose beads (Santa Cruz Biotechnology) and incubated with 1 μg of anti-ATG3 antibody (Sigma) or with control rabbit IgG (Santa Cruz Biotechnology) at 4° C overnight. Immune complexes were collected by adding 20 μl of protein AG-agarose beads equilibrated with lysis buffer. The immune complexes were washed three times with RIPA buffer, and proteins were resolved on a 4–12% Bis-Tris SDS-PAGE gel and transferred onto a PVDF membrane. The membranes were blocked with 1× blocking buffer and incubated with their respective primary (1:1000 dilution) and secondary antibodies (1:10000 dilution) as described above. Immunoreactive bands were visualized using a chemiluminescence system ECL.

### Microarray profiling

PC3 cells were transiently transfected with empty vector or TNFAIP8, and RNA was isolated using the RNeasy Mini Kit (QIAGEN) according to the manufacturer's instructions. The total RNA was pooled from three independent biological replicates. RNA concentration and purity (OD 260 nm/280 nm) were measured using a NanoDrop ND-1000 spectrophotometer (Thermo Fisher), and the RNA integrity number was determined using an Agilent 2100 Bioanalyzer Instrument (Agilent, Santa Clara, CA, USA). Gene expression profiling was performed using an Illumina HumanHT-12 v4 Expression BeadChip platform containing 47,000 probes that covered RefSeq and Unigene annotated genes (Illumina). Biotinylated cRNA was generated by labeling 500 ng total RNA using the Illumina TotalPrep-96 RNA Amplification Kit (Ambion) following the manufacturers’ instructions. Purified cRNA was quantified, and the fragment size was determined using an Agilent 2100 Bioanalyzer. Approximately 750 ng of biotinylated cRNA probe was hybridized overnight to the Illumina HumanHT-12 v4 Expression BeadChip. The chip was washed and scanned according to the manufacturer's instructions. The BeadChips were scanned using a HiScanSQ System (Illumina Inc., San Diego, CA, USA). Microarray images were registered and extracted automatically during the scan using the manufacturer's default settings. The array data were submitted to Array Express with accession number E-MTAB-6803.

### RNA isolation, cDNA Synthesis, and qPCR

PC3 cells were plated in 6-well plates at a density of 1 × 10^5^ cells/wells 24 h before transfection. Cells were transfected with 2 μg of empty pcDNA3.1 vector or TNFAIP8-Myc tagged plasmid DNA using lipofectamine-2000 transfection reagent (Invitrogen) according to the manufacturer's instructions. After 30 h of transfection, cells were harvested, and total RNA was isolated using the TRIZOL Reagent (Invitrogen, Carlsbad, CA, USA). RNA (1 μg) was reverse transcribed using a High Capacity cDNA Reverse Transcription kit (Applied Biosystems). cDNA was mixed with the Power SYBR Green PCR master mix (Applied Biosystems, Carlsbad, CA, USA) with both forward and reverse primers of TNFAIP8 and other cell cycle-related genes as indicated in (Supplementary Table 1). GAPDH was amplified as an internal control. The PCR mixtures were run on a QuantStudio 3 PCR System (Applied Biosystems) using relative quantitation according to the manufacturer's protocols.

### RT-PCR

Total RNA from PC3, MDA-MB-468, LNCaP, LLC1, THP-1, and SH-SY5Y cells were isolated using TRIzol reagent (Invitrogen). RNA (1 μg) was reverse transcribed using a High Capacity cDNA Reverse Transcription kit (Applied Biosystems). The expression of the five TNFAIP8 isoforms was analyzed using GoTaq^®^ Green PCR Master Mix (Promega). The following isoform-specific forward primers were used; Isoform 1: 5ʹ-CGAGTACATGTGAGCGGTAAT-3ʹ, Isoform 2: 5ʹ-AC CGAGAGAGCAGAGAACT-3ʹ, Isoform 3: 5ʹ-GGCTGT CCGGCTTCTTTAT-3ʹ, Isoform 4: 5ʹ- AGTCCATCCC TGTTGTGAATG-3ʹ, Isoform 5: 5ʹ-AAGTGCAGTGG TGAGATCATAG-3ʹ. We used a common reverse primer: 5ʹ-GATCTTCTCTGCCTCCTTCTTG-3ʹ (Figure 1A and Supplementary Figure 1A). PCR parameters were: 2 min denaturation at 95° C; 30 cycles of 95° C for 30 sec, 58° C for 30 sec, and 72° C for 40 sec; and a final extension at 72° C for 5 min. PCR was performed using a SimpliAmp thermal cycler (Applied Biosystem). The PCR products were electrophoresed on a 1.5% agarose gel, and gels were stained with ethidium bromide. The PCR-amplified bands were extracted from the agarose gel and sequenced to identify the isoforms.

### Plasmids and siRNA transfections

A human tumor necrosis factor alpha-induced protein 8, transcript variant 1 (TNFAIP8)-Myc-DDK-tagged ORF cDNA plasmid was obtained from Origene (Rockville- MD, Cat # RC202729). The plasmid encodes 198 amino acids of TNFAIP8 protein (isoform 1 or 3), with Myc tag (EQKLISEEDL), and a FLAG tag (DYKDDDDK). For transfection, PC3, LNCaP, and C4-2 prostate cancer cells were plated in 6-well plates at a density of 1 × 10^5^ cells/wells 24 h before transfection. Cells were transfected with 2–4 μg of empty pcDNA3.1 vector or TNFAIP8-Myc tagged plasmid DNA using lipofectamine-2000 transfection reagent (Invitrogen) according to the manufacturer's instructions. After 24–30 h of transfection, cells were harvested, and the expression of theTNFAIP8-Myc tagged protein was examined by immunoblotting. For siRNA transfection, we used to control and TNFAIP8 human siRNA purchased from Dharmacon (Lafayette, CO). PC3 cells were transfected with 100 nM control or TNFAIP8 siRNA using Lipofectamine RNAiMAX reagent (Invitrogen) according to the manufacturer's instructions. Following 30 h of transfection, cells were harvested and TNFAIP8 knockdown was confirmed by Western blotting using anti-TNFAIP8 antibody. Anti-GAPDH antibody was used as the control.

### Immunofluorescence

The EGFP-LC3B plasmid was obtained from Addgene (plasmid id-11546). PC3 cells (1 × 10^5^) were grown on coverslips in 6-well plates for 24 h and co-transfected with 2 μg of empty vector (pcDNA3.1) and EGFP-LC3B or TNFAIP8-Myc-tagged plasmid and EGFP-LC3B. The cells were simultaneously treated with 10 ng/ml TNFα (Invitrogen) and 3-methyladenine (4 mM) (Sigma) as indicated for 24 h. Cells were washed with PBS and fixed with 4% paraformaldehyde for 15 min. Cells were permeabilized with 0.25% Triton X-100 in blocking buffer (1% goat serum (Sigma) in PBS). Cells were washed twice with PBS and incubated with 1:500 dilutions of anti-Myc rabbit antibody in blocking buffer at 4° C for 18 h. Then, cells were washed twice with PBS and incubated with an Alexa-Fluor-568-conjugated anti-rabbit antibody (Invitrogen). Following immunostaining, cells were washed twice with PBS and mounted with Vectashield mounting medium (Vector Lab.) containing nuclear DAPI stain. Cells were imaged using an OlympusBX60 fluorescent microscope. The number of GFP-LC3B-related puncta formed in the cells (*n* = 10) was counted and plotted.

### TNFα and drug treatments

PC3, LNCaP, and C4-2 cells were grown in RPMI medium for 24 h to 70–80% confluency. The cells were treated with 10 ng/ml of TNFα for the indicated time periods, and the TNFAIP8 expression was analyzed by immunoblotting. For drug treatments, cells were treated with DMSO (control) or the indicated concentrations of doxorubicin (Fisher Scientific) and docetaxel (Selleckchem, Houston, TX, USA) for 24 h. Colony formation assays were performed as described below.

### Colony formation and cell proliferation

PC3, LNCaP, and C4-2 cells were grown in RPMI medium and transfected with TNFAIP8-Myc plasmid or pcDNA3.1 empty vector (2 μg/well in 6-well plates) for 24 h. After 24 h of transfection, cells were trypsinized and counted. Live cells were re-plated in 6-well plates (2000 cells/well) in triplicate and allowed to grow for 7–10 days. Cells were fixed with cold methanol and stained with 0.1% crystal violet for 30 min. Cells were washed with distilled water and allowed to dry. Blue colonies were counted and plotted. Cell proliferation was performed in 96-well plates using the WST-1 assay. PC3, LNCaP, and C4-2 cells were transfected with the TNFAIP8-Myc plasmid or empty vector for 18 h and treated with 0.5 nM of docetaxel or 1 μM doxorubicin for 24 h. Then, cells were trypsinized and counted. Live cells were re-plated in 96-well plates (1 × 10^4^ cells/well) and incubated for 48 h. Cell proliferation was measured by adding 10 μl WST-1 reagent according to manufacturer's instructions (Roche Applied Science, Indianapolis, IN, USA). Cell proliferation was measured at 450 nm using Fluostar Omega plate reader (BMG Lab tech, Cary, NC, USA).

### Statistical analysis

The data from cell colony formation and cell proliferation were analyzed using a two-tailed Student's *t*-test. Data are expressed as the mean ± standard deviation (S.D.). *p* values are shown in figures.

## SUPPLEMENTARY MATERIALS FIGURES AND TABLES


