# Supplementary material for: TNFAIP8 promotes prostate cancer cell survival by inducing autophagy
Source: Oncotarget. 2018 Jun 1;9(42):26884–99. doi: 10.18632/oncotarget.25529 (PMC6003558; doi:10.18632/oncotarget.25529)
Supplement: Supplementary file 1 [file oncotarget-09-26884-s001.pdf]

TNFAIP8 promotes prostate cancer cell survival by inducing autophagy

SUPPLEMENTARY MATERIALS

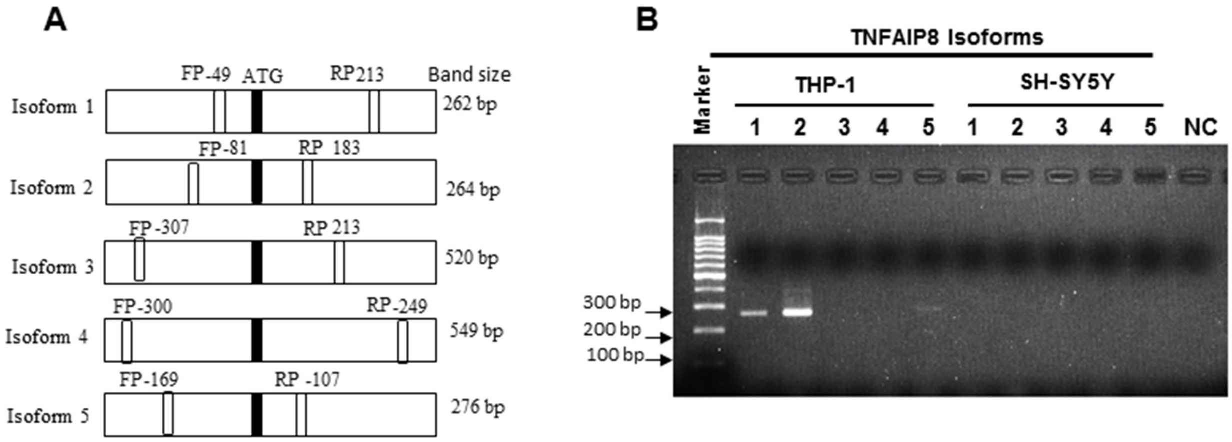

Supplementary Figure 1: Designing the forward and reverse primers to analyze the expression of TNFAIP8 isoforms. **(A)** A schematic of the forward and reverse primer design process. (**B)** RT-PCR of total RNA from human monocytic leukemia THP-1 cells and human neuroblastoma SH-SY5Y cells. RNA of the five isoforms was amplified, and PCR products were electrophoresed on a 1.5% agarose gel stained with ethidium bromide. NC–negative control (no cDNA).

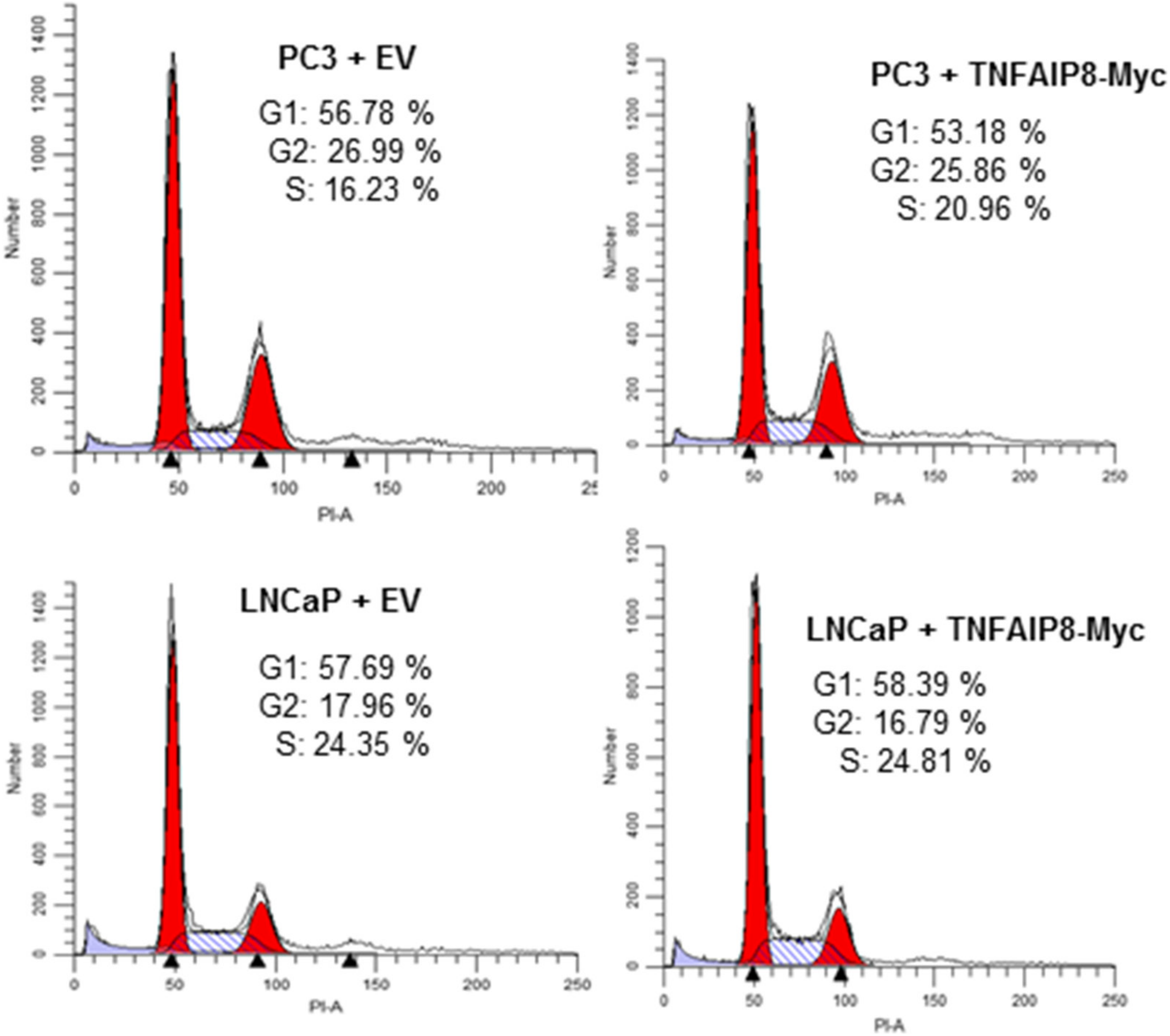

Supplementary Figure 2: The effect of TNFAIP8 expression on cell cycle progression. PC3 and LNCaP cells were transfected with empty vector or TNFAIP8-Myc plasmid for 30 h. Cells were stained with propidium iodide, and cell-cycle progression was monitored using flow cytometry. The percentages of cells in the G1, G2, and S phases were measured.

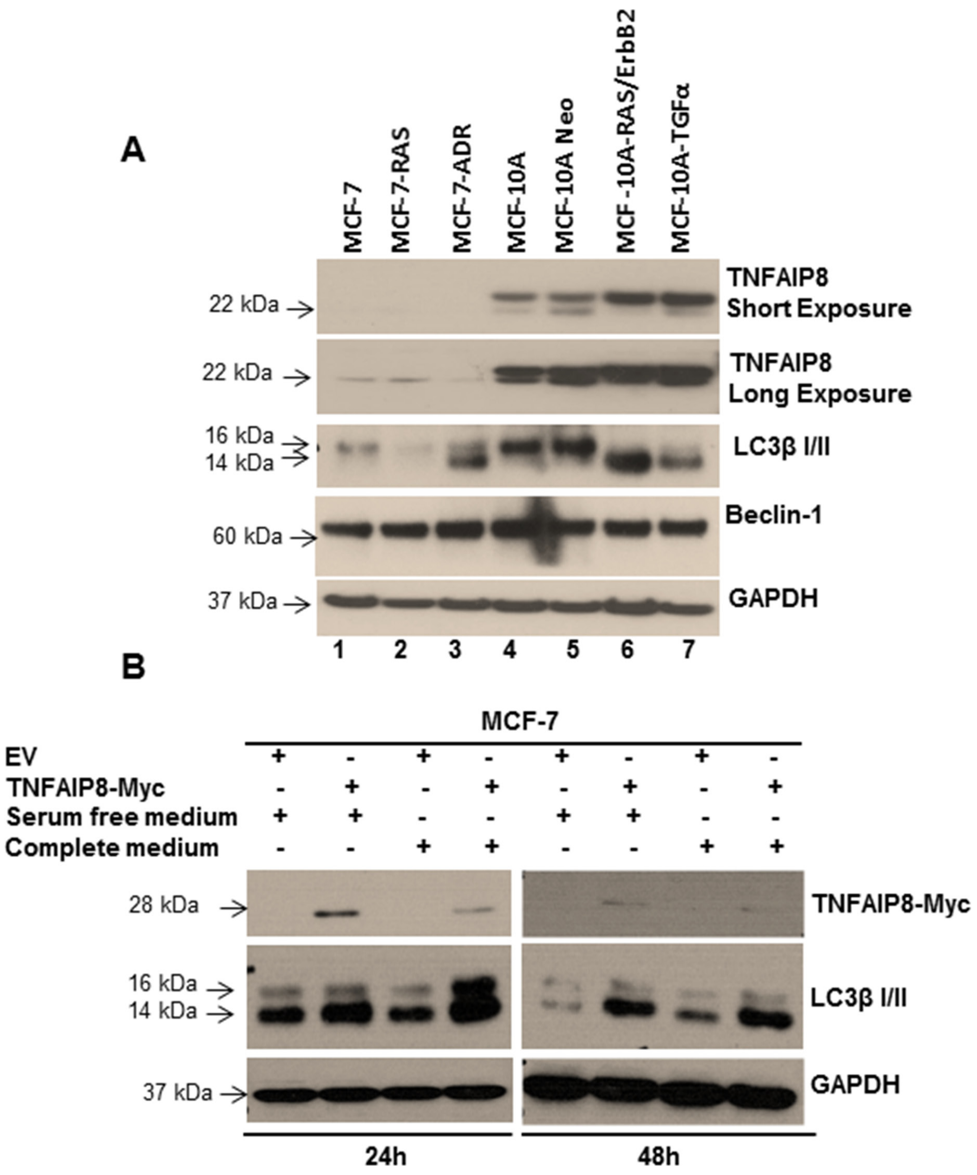

Supplementary Figure 3: TNFAIP8 expression is associated with LC3 β I/II expression in normal and breast cancer cells. (A) MCF7, MCF7-RAS, MCF7-ADR, MCF10A, MCF10A-Neo, MCF10A-RAS, MCF-10A-TGFα cells were grown for 30 h and cell lysates were immunoblotted with anti-TNFAIP8, anti-LC3β I/II, anti-Beclin1, and anti-GAPDH antibodies. (**B)** MCF7 cells were transfected with empty vector or TNFAIP8-Myc plasmid. After 18 h, the medium was changed to serum-free (SF) medium or complete medium for an additional 24 h and 48 h. Cell extracts (40 µg) were immunoblotted with anti-Myc, anti-LC3β I/II, and anti-GAPDH antibodies as indicated.

Supplementary Table 1: The following primer sets were used for RT/qPCR analysis of TNFAIP8 and cell cycle-related gene expression

TNFAIP8 FP: 5′- CTTTGACCGGAATGTGTTATCCA -3′<br>TNFAIP8 RP: 5′- CAAGGCAGCCAAAAATTCACAA -3′
CDK2 FP: 5′- CCAGGAGTTACTTCTATGCCTGA -3′<br>CDK2 RP: 5′- TTCATCCAGGGGAGGTACAAC -3′
CDC20 FP: 5′- GCACAGTTCGCGTTCGAGA -3′<br>CDC20 RP: 5′- CTGGATTTGCCAGGAGTTCGG -3′
MAD2L1 FP: 5′-GGACTCACCTTGCTTGTAACTAC -3′<br>MAD2L1 RP: 5′-GATCACTGAACGGATTTCATCCT -3′
CDT1 FP: 5′- CGGTGGACGAGGTTTCCAG -3′<br>CDT1 RP: 5′-CTGCCGGGGTGGATTTCTT -3′
PCNA FP: 5′- ACACTAAGGGCCGAAGATAACG -3′<br>PCNA RP: 5′- ACAGCATCTCCAATATGGCTGA -3′
CHEK1 FP: 5′- ATATGAAGCGTGCCGTAGACT -3′<br>CHEK1 RP: 5′- TGCCTATGTCTGGCTCTATTCTG -3′
CCNE2 FP: 5′- TCAAGACGAAGTAGCCGTTTAC -3′<br>CCNE2 RP: 5′- TGACATCCTGGGTAGTTTTCCTC -3′
CCNB2 FP: 5′-TGCTCTGCAAAATCGAGGACA -3′<br>CCNB2 FP: 5′-GCCAATCCACTAGGATGGCA -3′
CCNA2 FP: 5′- CGCTGGCGGTACTGAAGTC -3′<br>CCNA2 RP: 5′- GAGGAACGGTGACATGCTCAT -3′
CDCA4 FP: 5′- GAGATGACGCAGGATGGGAC -3′<br>CDCA4 RP: 5′- TCCACGTCTGAGAACAGCTCT -3′
CDC45 FP: 5′- GCACAGTTCGCGTTCGAGA -3′<br>CDC45 RP: 5′- CTGGATTTGCCAGGAGTTCGG -3′
GAPDH FP: 5′- CCACCCAGAAGACTGTGGAT -3′<br>GAPDH RP: 5′- GTTGAAGTCAGAGGAGACCACC -3′
